# Impact of IPSS-M implementation in real-life clinical practice

**DOI:** 10.3389/fonc.2023.1199023

**Published:** 2023-05-18

**Authors:** Irene Zamanillo, Maria Poza, Rosa Ayala, Inmaculada Rapado, Joaquín Martinez-Lopez, Maria Teresa Cedena

**Affiliations:** Hematology Department and Research Institute (imas12), University Hospital 12 Octubre, Madrid, Spain

**Keywords:** IPSS-M, myelodysplastic syndrome, prognostic score, real-life, validation, risk

## Abstract

**Objectives:**

The IPSS-M is a recently published score for risk stratification in myelodysplastic syndromes (MDS), based on clinical and molecular data. We aimed to evaluate its relevance on treatment choice in a real-life setting.

**Methods:**

We retrospectively collected clinical, cytogenetic and molecular data from 166 MDS patients. We calculated IPSS-R and IPSS-M scores and compared Overall Survival (OS) and Leukemia Free Survival (LFS). We also analyzed which patients would have been affected by the re-stratification in terms of clinical management.

**Results:**

We found that 86.1% of the patients had at least one genetic alteration. The most frequent mutated genes were *SF3B1* (25.9%), *DNMT3A* (16.8%) and *ASXL1* (14.4%). IPSS-M re-stratified 48.2% of the patients, of which 16.9% were downgraded and 31.3% were upgraded. IPSS-M improved outcome prediction, with a Harrell’s c-index of 0.680 vs 0.626 for OS and 0.801 vs 0.757 for LFS. In 22.2% of the cohort, the reclassification of the IPSS-M could potentially affect clinical management; 17.4% of the patients would be eligible for treatment intensification and 4.8% for treatment reduction.

**Conclusions:**

IPSS-M implementation in clinical practice could imply different treatment approaches in a significant number of patients. Our work validates IPSS-M in an external cohort and confirms its applicability in a real-life setting.

## 1 Introduction

Myelodysplastic syndromes (MDS) are a heterogeneous group of clonal hematopoietic neoplasms, characterized by cytopenias, bone marrow dysplasia and increased risk of acute myeloid leukemia transformation (1).

From a clinical point of view, the evolution of MDS can be greatly variable, depending on both disease and patient-related factors. Therefore, identifying high-risk patients is of great concern when deciding the best treatment for each patient. Traditionally, the IPSS-R score has been employed to predict patient outcome. This score is based on clinical and analytical data, as well as cytogenetic alterations, and has been widely validated (2–5). In recent years, the development of new technologies has led to a great improvement in our knowledge of the genetic landscape in MDS. Multiple studies have investigated the role of somatic mutation in MDS patient outcome (6–11).

The IPSS-M is a recently developed score for risk stratification in MDS, which incorporates molecular data to clinical and analytical parameters. It classifies patients in six categories, leading to re-stratification of 46% of the patients and improved outcome prediction for both overall survival (OS) and leukemia free survival (LFS) (12).

A few studies have validated this prognostic score in an external cohort. Sauta et al. reported improved discrimination of IPSS-M compared to IPSS-R for OS and LFS, and proved its applicability in post-allo stem cell transplantation setting (13). Baer et al. confirmed these results and performed an exploratory analysis on the importance on individual genes for risk prediction, since some mutations included in the score, such as *MLL-PTD*, may not be routinely analyzed in all laboratories (14).

However, there is still scarce evidence on the impact of IPSS-M application on patient management. The nature of the classification in six progressive risk categories means that some re-stratifications will not be translated into different clinical approaches. For example, patients increasing from very low to low risk will still be eligible for low-risk treatment strategies. Furthermore, some patients are not candidates for aggressive therapies due to old age and/or comorbidities, and even a significant risk re-stratification would not mean a difference in patient management.

In this study we aimed to analyze the impact of IPSS-M application on treatment choice in a real life-setting.

## 2 Materials and methods

We conducted a retrospective study in which we identified 166 MDS patients diagnosed between 2001 and 2022 at our institution. We also included secondary MDS and MDS/NMPC overlap syndrome patients, as they are also included in the initial IPSS-M publication. This study was approved by the Hospital 12 de Octubre Institutional Review Board as part of the CRIS project against cancer.

We collected clinical and analytical data from their medical record. Diagnostic studies were conducted on bone marrow samples. Cytogenetic data were obtained from FISH and G-banding examination. An in-house NGS panel including 44 frequently mutated myeloid genes was performed at diagnosis in all the patients, lacking a few genes included in the IPSS-M (*ETKN1, GATA2, GNB1, PPM1D, PRPF8* and *PTPN11*). *MLL PTD* was not tested in the patients.

IPSS-R and IPSS-M categories were calculated for all the patients. In order to compare reclassification between the two risk scores, we merged moderate-low and moderate-high IPSS-M risk categories into a single intermediate group. When examining re-stratification impact on treatment choice, we divided patients in three risk groups (very low-low, intermediate and high-very high). Given the limited treatment options approved for MDS and following the current treatment guidelines, further risk discrimination was not considered clinically significant.

IBM IPSS^®^ V25 and Python were used for the statistical analysis. Mean and standard deviation (SD) were used for homogeneous distribution measures and median and interquartile range (IQR) for those of asymmetric distribution. We used *Harrell’s* concordance index (c-index) to evaluate the prediction accuracy s IPSS-R and IPSS-M. Survival analysis was performed using the Kaplan-Meier method and comparisons between groups were assessed using the log-rank test. Two-sided p values of <0.05 were considered statistically significant.

## 3 Results

A total of 166 patients were included in the analysis. The median age at diagnosis was 65 years (IQR 68-82) and 34.3% pf the patients were female.

The most frequent entities according to WHO 2017 classification were refractory cytopenia with multilineage dysplasia (24.7%), myelodysplastic syndrome with excess blasts I (17.5%) and refractory cytopenia with multilineage dysplasia and ring sideroblasts (14.5%) (**Table 1**). A 9.6% of the patients presented with secondary/therapy related MDS and a 13.8% fulfilled diagnostic criteria for MDS/NMPC overlap syndromes.

**Table 1:** Patient diagnosis according to WHO 2017.

Entity	Frequency: n (%)
Refractory cytopenia with unilineage dysplasia	7 (4.2)
Refractory anemia with ring sideroblasts	10 (6)
Refractory cytopenia with multilineage dysplasia	41 (24.7)
Refractory cytopenia with multilineage dysplasia and ring sideroblasts	24 (4.5)
Myelodysplastic syndrome with excess blasts I	29 (17.5)
Myelodysplastic syndrome with excess blasts II	22 (13.3)
Myelodysplastic syndrome 5q-	7 (4.2)
Myelodysplastic syndrome unclassifiable	1 (0.6)
Chronic myelomonocytic leukemia	21 (12.7)
Overlap MDS/MPS	4 (2.4)

### 3.1 Genetic landscape

**Figure 1:**
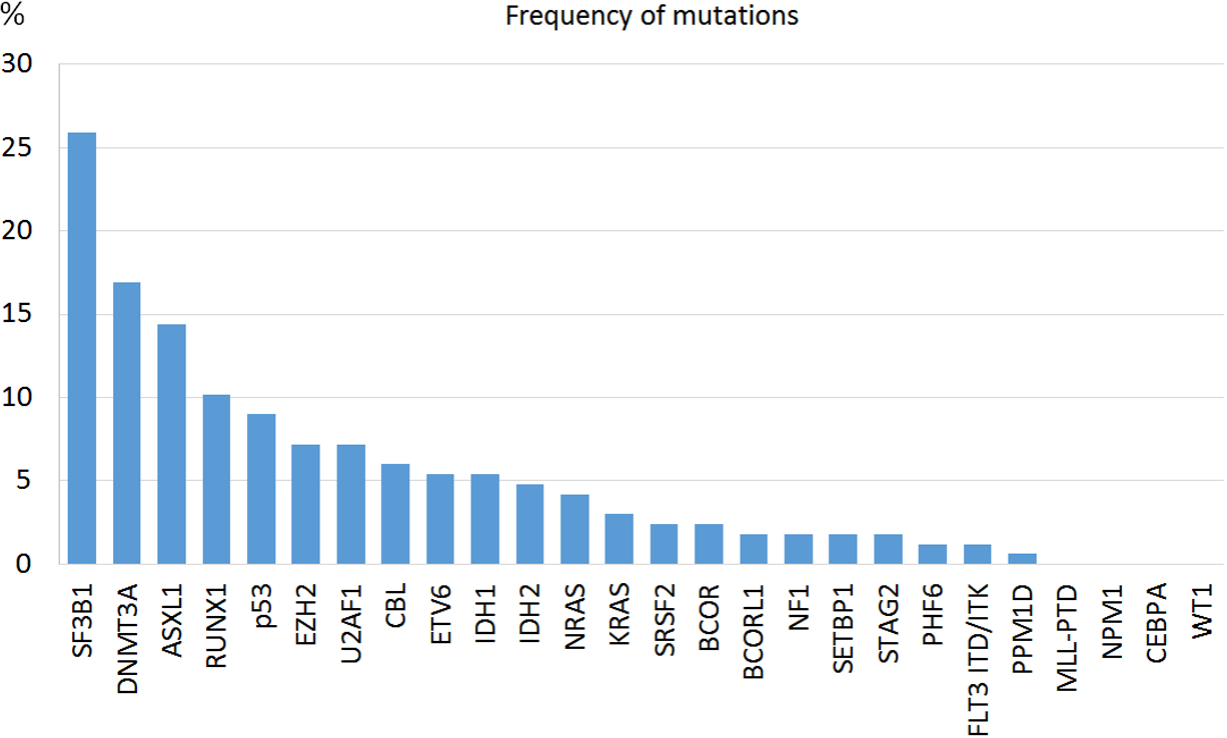
Frequency of mutations found in MDS patients at diagnosis.

In 86.1% of the patients we could identify at least one genetic alteration: 34.3% presented only somatic mutations, 12% only cytogenetic abnormalities and 39.8% both. The most common mutated genes were *SF3B1* (25.9%), *DNMT3A* (16.8%) and *ASLX1* (14.4%) (**Figure 1**). *TP53* was mutated in 15 patients (9%). All mutations were more frequent in the IPSS-M high or very high risk groups except for SF3B1 mutation (**Table 2**). Median and mode number of somatic mutations per patient was 1, and 15% of the patients presented three or more NGS mutations. The mean number of mutations per patient was 0.81 (SD 0.76) for IPSS-M very low/low, 1.44 (SD 1.11) for IPSS-M moderate low/moderate high and 2.15 (SD 1.47) for IPSS-M high/very high. Recurrent cytogenetic alterations were similar to those previously described in MDS, with del(5q) in 22 patients (13.3%), -7/del(7q) in 14 (8.4%), complex karyotype in 18 (10.8%) and del17p/-17 in 3 patients (1.8%).

**Table 2:** Frequency of mutations in the different IPSS-M categories.

		Very low/low (%)	Moderate low/moderate high (%)	High/very high (%)
**RNA Splicing**	SF3B1	27	23,2	13
	SRSF2	1,3	2,3	4,3
	U2AF1	2,6	9,3	10,8
**DNA Methylation**	DNMT3A	11,7	13,2	19,5
	IDH1	3,9	9,3	4,3
	IDH2	2,6	4,6	8,7
**Chromatin modification**	ASLX1	9,8	20,9	21,7
	EZH2	1,3	9,3	15,2
**Transcription regulation**	RUNX1	2,6	6,9	26,1
	ETV6	1,3	11,6	6,5
**DNA repair**	p53	2,6	0	28,2
**Signal transduction**	CBL	1,3	11,6	8,7
	NRAS	1,3	2,3	10,8
	KRAS	1,3	6,9	2,1

### 3.2 Risk stratification and patient outcome

**Figure 2:**
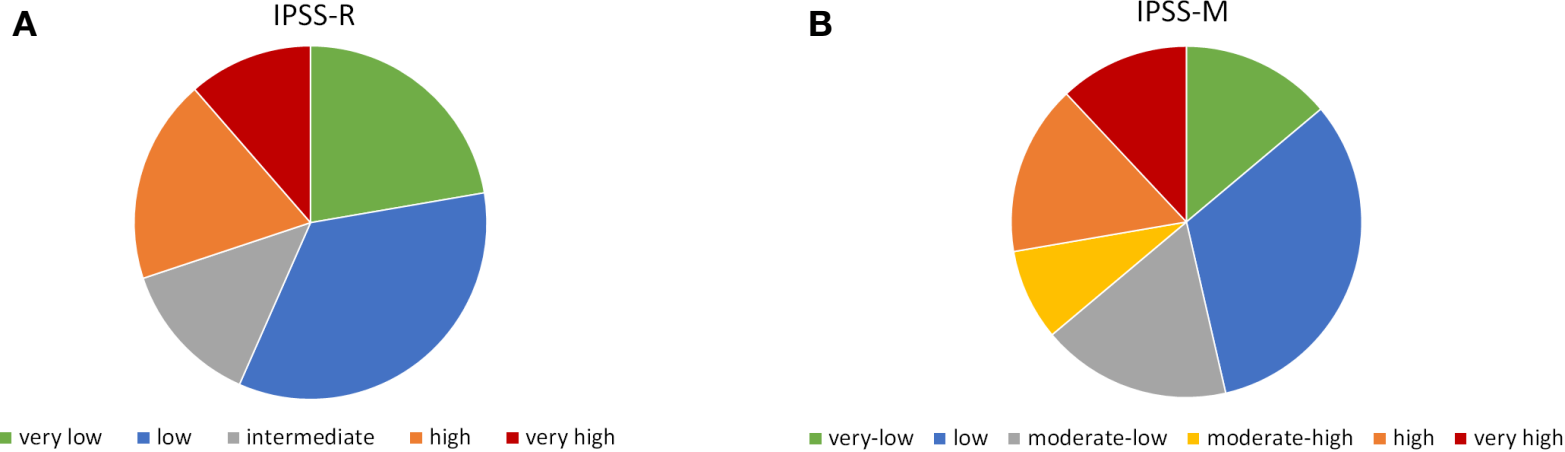
Risk distribution according to IPSS-R **(A)** and IPSS-M **(B)**.

Patient distribution according to IPSS-M categories was 13.9% very low, 32.5% low, 17.5% moderate-low, 8.4% moderate-high, 15.7% high and 12% very high, which is similar to previous reports. For IPSS-R distribution was 22.3% very low, 34.3% low, 13.3% intermediate, 18.7% high and 11.4% very high (**Figure 2**).

**Figure 3:**
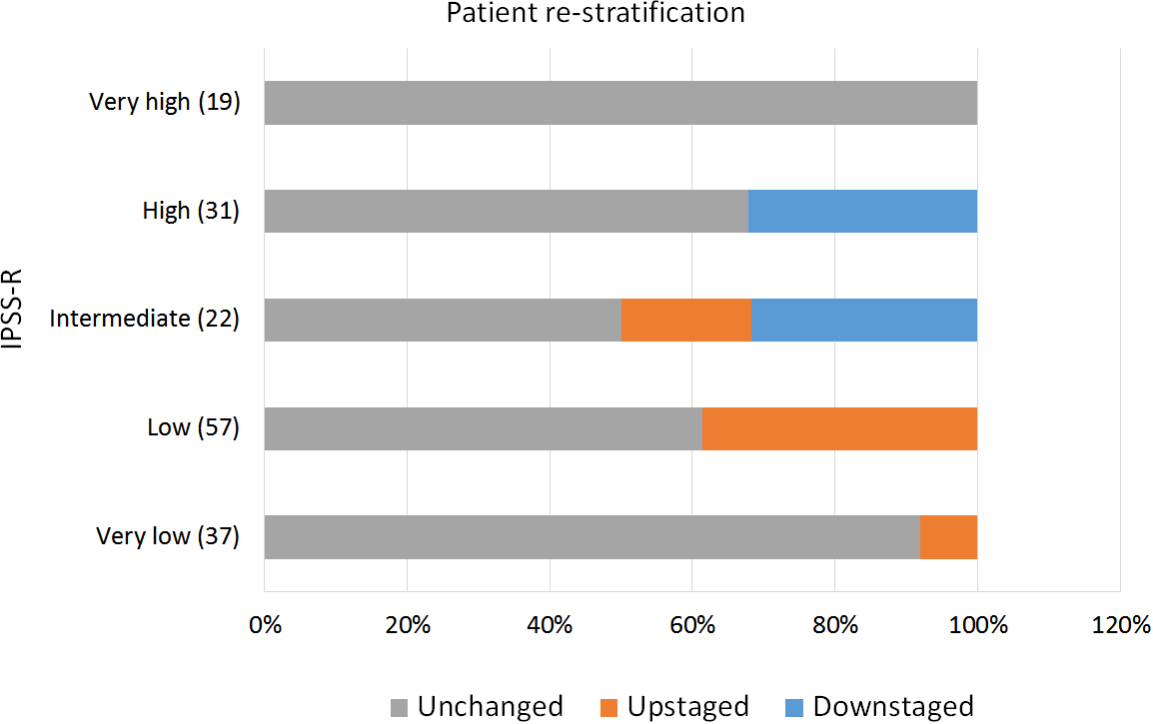
Risk re-stratification for patients in different IPSS-R risk categories.

IPSS-M re-stratified 48.2% of the patients, of which 16.9% were down-staged and 31.3% were up-staged. Only 7 patients (4.2%) shifted more than one risk strata (e.g. from intermediate to very high). Interestingly, 50% of the patients among the IPSS-R intermediate group changed to a different risk category, 18.2% to a lower one and 31.8% to a higher one (**Figure 3**).

**Figure 4:**
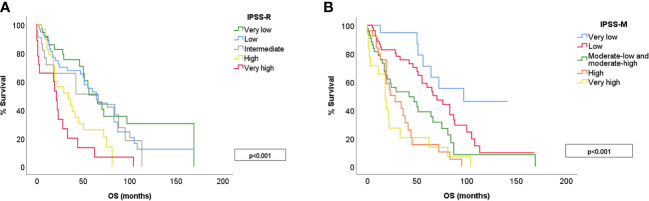
OS for different risk categories in IPSS-R **(A)** and IPSS-M **(B)**. IPSS-M 3 includes moderate-low and moderate-high risk groups.

The median survival of the global cohort was 50 months with a median follow-up of 25.5 months. Twenty patients (12%) suffered a leukemic transformation. IPSS-M improved patient outcome prediction for both OS and LFS, showing a clear discrimination between groups (**Figure 4**). We calculated *Harrell’s* c-index to determine the correlation between each score prediction and the real outcome. IPSS-R c-index was 0.626 for OS and 0.757 for LFS. IPSS-M improved both predictions, obtaining a c-index of 0.680 for OS and 0.801 for LFS.

### 3.3 Treatment

During the course of the disease, 69.3% of the patients required treatment. A 39% were treated with erythropoietin and 22.9% received hypomethylating agents. Five patients received an allo-stem cell transplantation (SCT).

**Figure 5:**
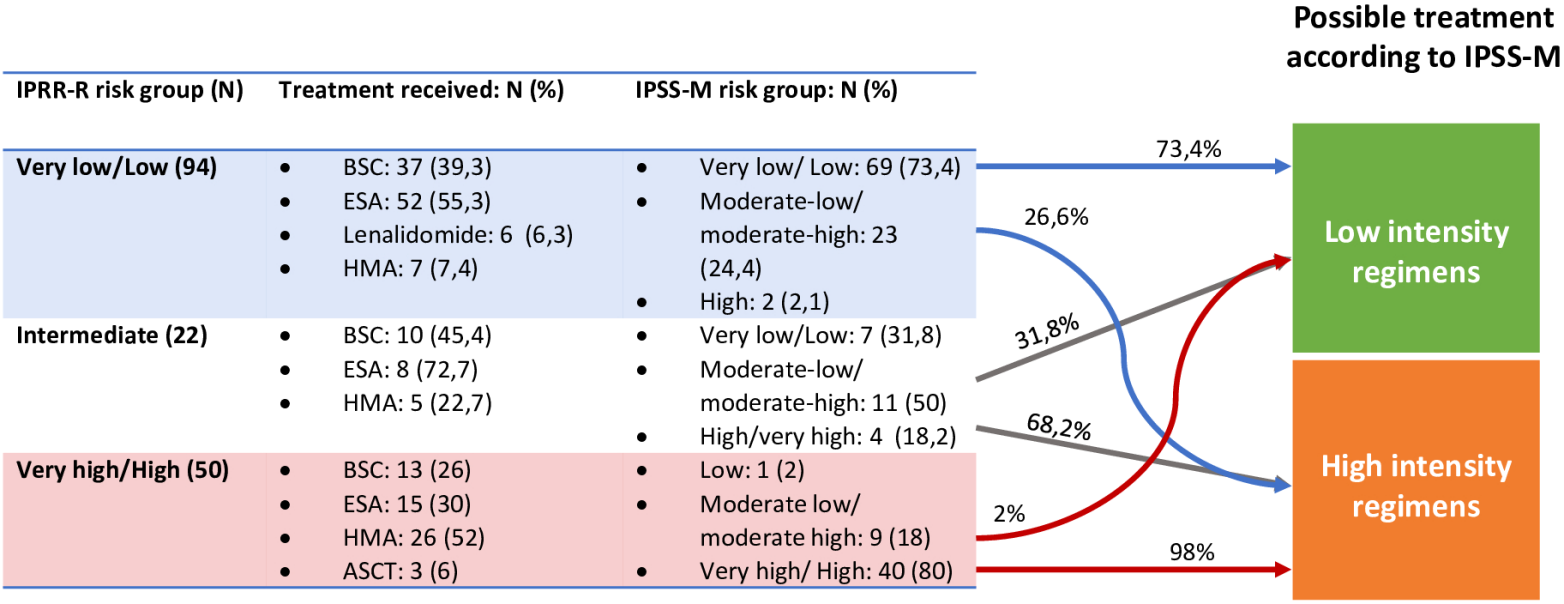
Re-stratifications and treatment impact. In the fourth column is represented the treatment the patients could have received if IPSS-M classification had been studied at diagnosis. ASCT, Allogenic Stem Cell Transplantation; BSC, Best supportive care; ESA, Erytropoiesis stimulating agents; HMA, Hypomethylathing agents.

We then analyzed patient re-stratification implication on treatment selection. The current guidelines stablish different recommendations for low-risk and high-risk patients. Therefore, when analyzing risk reclassification importance on patient therapy, we considered only three risk groups: very low-low, intermediate and high-very high, and risk up or down-staging was only considered relevant when it implied a shift between these three categories. In our cohort IPSS-M reclassification would potentially affect treatment choice in 22.2% of the patients; 17.4% (29) would be eligible for treatment intensification and 4.8% (13) for lower intensity regimens (**Figure 5**).

Finally, we performed an individual examination of the 29 up-staged cases who would have been eligible for more intensive therapies. Among these patients, only 11 would have received a different therapy. Eighteen would not have been candidates to a higher intensity regimen, due to age, comorbidities or ECOG. This represents a 6.6% of the global cohort.

## 4 Discussion

In this study, we calculated both IPSS-R and IPSS-M score for 166 patients with *de novo* MDS at our center. The IPSS-M showed a clear separation of the different risk categories for both OS and LFS, and outperformed IPSS-R in the correlation analysis (c-index was 0.680 for OS and 0.801 for LFS). This is in line with previously published results (13–15), which confirm a more accurate risk prediction with IPSS-M for both LFS and OS. This growing body of evidence confirms the power of IPSS-M to accurately predict MDS patients outcome, improving current scores.

Interestingly, these results were obtained even though our NGS panel was missing several genes included in the IPSS-M score. This can probably be justified by the low frequency of these mutations in MDS, and confirms the applicability of the score even with some missing data. This is of great importance, as some of the mutations from the original publication are not routinely tested for clinical practice. Other papers have studied the impact of missing genes on IPSS-M. Sauta et al. (13) found an 80% accuracy with a set of 15 genes, but it decreased to <70% and <60% with 10 and 7 genes respectively. Baer et al. (14) found the determination of p53, KMT2A PTD and FLT3 essential for the determination of IPSS-M, but since the frequency of FLT3 and KMT2A PTD mutations are very low (around 1%) their absence affects a low proportion of cases.

Mutation distribution in our series matched the one described by Cazzola et al. (16). Mutations across all biological pathways were more frequent in high-risk patients except for SF3B1 mutation, which is associated with refractory anemia with ring sideroblasts and appeared in the low risk group. As expected, the mean number of mutations per patient was progressively higher as the risk increased (0.81 in low risk, 1.44 in moderate risk and 2.15 in high risk).

The application of IPSS-M reclassified a significant number of patients (48.2%), most of them to a higher risk category. This re-stratification was of only one step in most of the cases, which is understandable since IPSS-R and IPSS-M share some of the parameters. Several publications have explored (13–15, 17, 18) the role of IPSS-M in patient re-stratification with comparable results. Sauta et al. (13) validated the score in a European cohort including 2876 MDS patients from 21 centers. In this study, IPSS-M re-stratified 46% of the patients, 23.6% were upstaged and 22.4% were downstaged. Aguirre et al. (15) implemented the score in an American population treated at the Moffit Center, in which 45% of the patients were re-classified. Baer et al. (14) also reported a 44% of re-stratifications in a German cohort. This stable results in different cohorts speaks to the stability of the molecular landscape in different populations.

We then focused on IPSS-M impact on treatment choice for MDS patients. Current treatment guidelines for MDS are based on patient age and comorbidities, as well as disease risk. In general, erythropoietin stimulating agents (ESA) or lenalidomide are recommended for lower risk disease while hypomethylating agents or allo-SCT with or without previous chemotherapy are recommended for higher risk disease, if patients are eligible for this therapy. Intermediate-risk patients can either follow high or low risk strategies, depending on clinical manifestations of the disease and patient baseline status. Therefore, not all re-stratifications would have meant a difference in clinical management for the patients, for example, patients changing from very low to low-risk would still be considered for low risk strategies.

To analyze IPSS-M implications on patient management we considered only three risk groups: low (including very low and low risk patients), intermediate and high (including high and very high-risk patients). In our cohort, 22.2% of the patients could have been managed differently according to IPSS-M. A 17.4% would have been considered for high-risk therapies and 4.8% for low risk therapies. However, MDS is frequent in advanced age patients who frequently suffer from other pathologies and not all high-risk patients may be eligible for intensive therapies. In our cohort, 11 of the 29 up-staged patients could potentially have received a more intensive treatment due to a poor performance status or comorbidities. To our knowledge, few studies have studied treatment impact of IPSS-M utilization. Novoa Jauregui et al. (19) studied IPSS-M impact on patient management and found a similar proportion of patients with “treatment relevant” re-stratifications (17.4%), 11.9% up-staged and 5.6% downstaged.

Regarding IPSS-M applicability in different treatment settings, Sauta et al. (13) found the score improved relapse and survival prediction after ASCT, as well as OS for HMA treated patients. However, it did not predict response to HMA. In our cohort of patients treated with HMA 5/11 patients responded in the low and moderate risk group, and 7/22 in the high risk group, with no statistical difference.

This study presents some limitations due to the retrospective design and moderate number of patients analyzed. However, we consider that the cohort included in the study is representative of the highly variable MDS population. Besides, the results obtained were robust in regards of the improved outcome prediction with IPSS-M compared to IPSS-R despite these limitations.

In conclusion, IPSS-M should be the new standard for risk stratification in MDS, as it provides a more accurate risk prediction than IPSS-R- The score provides reliable results even if not all somatic mutations included in the score are tested. This study validates initial findings in real life cohort.

Importantly, IPSS-M implementation in clinical practice could potentially affect treatment choice in a significant number of patients. In our cohort, 22.2% could have received a different treatment if IPSS-M risk had been considered at diagnosis.

## Data availability statement

The raw data supporting the conclusions of this article will be made available by the authors, without undue reservation.

## Ethics statement

The studies involving human participants were reviewed and approved by Hospital 12 de Octubre Institutional Review Board. The patients/participants provided their written informed consent to participate in this study.

## Author contributions

MTC conceived and designed the analysis. IZ and MP collected the data. IZ performed the analysis and wrote the article. RA, IR, and JM-L supervised and revised the manuscript. All authors contributed to the article and approved the submitted version.
